# The prevalence of ADSL (rs3788579) and CYP1A2 (rs17861162) polymorphisms in female breast cancer patients in North-West Iran

**DOI:** 10.1007/s12672-024-00919-z

**Published:** 2024-03-03

**Authors:** Mohammad Valizadeh Osalo, Parisa Hosseini, Hamed Charkhian, Hossien Soltanzadeh, Selda Goharkhany, SEREF BUGRA TUNCER

**Affiliations:** 1 Department of Genetics, Bonab Branch, Islamic Azad University, Bonab, Iran; 2 Department of Biotechnology, Urmia Branch, Islamic Azad University, Urmia, Iran; 3 Young Researchers Club, Urmia Branch, Islamic Azad University, Urmia, Iran; 4 Medicinal Plants Research Center, Maragheh University of Medical Sciences, Maragheh, Iran; 5 Department of Biology, Faculty of Natural Science, University of Tabriz, Tabriz, Iran; 6 Department of Cancer Genetics, Institute of Graduate Studies in Health Sciences, Istanbul University, Istanbul, Turkey

**Keywords:** ADSL, CYP1A2, Polymorphism, Breast cancer

## Abstract

**Introduction:**

Breast cancer is a prevalent and significant contributor to cancer-related mortality among women worldwide. Its increasing incidence, especially in regions like North-West Iran, necessitates a deeper understanding of genetic factors contributing to its development. Genetic alterations, particularly single nucleotide polymorphisms (SNPs), are implicated in breast cancer susceptibility, making investigation in this context crucial. This study explores the role of CYP1A2-rs17861162 and ADSL-rs3788579 SNPs in breast cancer risk among Iranian women.

**Methods:**

This study involved 200 female breast cancer patients and 200 healthy controls in North-West Iran. DNA was extracted from blood samples, and PCR–RFLP was used for genotyping the CYP1A2 and ADSL genes.

**Results:**

The CYP1A2-rs17861162 SNP exhibited a shift from the C allele to the G allele in breast cancer patients, resulting in a 21.7% decrease in CC genotype frequency and a 21.6% and 77.8% increase in CG and GG genotypes, respectively, compared to controls. In ADSL-rs3788579 SNP, breast cancer patients had a significantly higher prevalence of the T allele, with a 28.5% increase compared to controls. In healthy participants, CC was most common, while in the breast cancer group, TT was most common.

**Conclusion:**

This study highlights significant genetic alterations in CYP1A2-rs17861162 and ADSL-rs3788579 SNPs among breast cancer patients in North-West Iran, suggesting their potential as diagnostic and prognostic biomarkers. Further research is warranted to elucidate the precise mechanisms underlying their contributions to breast cancer susceptibility in this population.

## Introduction

Breast cancer is now the most commonly diagnosed cancer worldwide, constituting approximately 24.5% of all cancer cases [1, 2]. t also accounts for 15.5% of cancer-related deaths, making it a significant contributor to cancer-related mortality in women. This is largely due to the complexity of the disease, including its various manifestations, capacity to spread, and resistance to treatment [1, 3, 4]. Despite extensive research efforts spanning several decades encompassing laboratory work, epidemiological studies, and clinical investigations, the incidence of breast cancer has continued to rise for most of the past forty years. In the recent decade (2010–2019), there was an annual increase in the incidence rate of 0.5% [5, 6]. Recent studies report an increase in incidence in the Asian continent, including Iran [7]. Currently, breast cancer is the most common neoplastic disease among Iranian women, with an age of onset 10 years below the global average [8, 9].

Breast cancer is a considerably heterogeneous disease with numerous genetic and environmental risk factors [10]. Genetic alterations (mutations or omissions) to the DNA structure may result in significant cellular damage or cancerous changes [11]. Approximately 5–10% of all instances of breast cancer arise from familial and hereditary causes [12]. Studies also report an increased risk for developing breast cancer in individuals with a genetic predisposition who are also exposed to environmental risk factors [13]. As the most frequently occurring genomic alterations, single nucleotide polymorphisms (SNPs) are responsible for the majority of genomic diversity and subsequent inter-personal phenotypic variation [14, 15]. Furthermore, SNPs also act as predisposing genetic factors, contributing to the development and progression of cancers [14], including breast cancer [16].

Cytochrome (CY) enzyme family is a mega-family consisting of more than a hundred proteins all containing a heme group and overall structural resemblance. Cytochromes can be found in a wide range of living organisms, albeit with minor differences [17]. Cytochrome P450 proteins refer to a superfamily of complex monooxygenases that catalyze various substrates (e.g. xenobiotics, estrogen, testosterone, cholesterol, vitamin D, steroids, fatty acids, bile acids, and numerous exogenous compounds, including drugs and toxins) [18]. CYP1 is a prominent member of P450s, comprising an extensive genomic group responsible for mitigating the biological effects of various chemical compounds. CYP1 itself is comprised of three sub-groups: A, B, and C, with CYP1A comprising three members (CYP1A1 through 3) [19].

CYP1A2 corresponds to the metabolic enzymes active in phase I reactions of cytochrome P450 and is responsible for the expression of 15% of P450 proteins. Although it was previously believed that CYP1A2 is exclusively produced by the liver [20], recent studies have also identified the breasts as a secondary production site [21]. CYP1A2 is located on chromosome 15, with a size of 7.8 kb and comprising 7 expressional and one non-coding exon [22]. This enzyme is directly involved in the metabolism and neutralization of 10–13% of all medications and carcinogens [22, 23]. The expression and activity of CYP1A2 are heavily influenced by genetic polymorphism [10, 24]. Several studies report an association between CYP1A2 and lung and bladder cancers, while its relationship with breast cancer in different ethnicities needs further investigation [25]. To date, researchers have identified 150 variant alleles for this gene, of which 26–30 are considered nonsynonymous polymorphisms with a frequency of less than 1% [22, 23].

The metabolism of purine nucleotides has always been of clinical interest [26]. Adenylosuccinate lyase (ADSL, EC 4.3.2.2) is a bi-functional enzyme that catalyzes the de novo conversion of Phosphoribosylaminoimidazolesuccinocarboxamide (SAICAR) to 5-Aminoimidazole-4-carboxamide ribonucleotide (AICAR) and adenylosuccinate (S-AMP) to adenosine monophosphate (AMP) at the inosine branch point of the purine biosynthetic pathway, as presented below [27, 28]:

1. 5-aminoimidazole-4-N-succinocarboxamide ribotide to 5-aminoimidazole-4-carboxamide ribotide + fumarate.
2. adenylosuccinate to 5'-adenylic acid + fumarate.

ADSL also plays a prominent role in the structure of DNAs and RNAs, metabolism, cell survival, and proliferation [29, 30]. The importance of purine biosynthesis pathway enzymes first came to notice after studies on severe mental retardation, autistic traits, and conditions such as Down syndrome and sensorineural deafness [26]. In cancer cells, a significant imbalance can be seen between the purine metabolism enzymatic pattern and cellular alteration and cancer progression [31]. Several investigations have reported reduced adenylosuccinate lyase levels in various neoplasias, such as transplanted tumors, transformed cell lines, and human tumors [32]. The ADSL encoding gene is located on chromosome 22q13.1 → q13.2, and defects in its corresponding enzyme are considered genetically heterogeneous [26]. To date, over 50 ADSL mutations affecting protein biogenesis, structural stability, activity, and purinosome assembly have been identified [33]. Recently, the results of a genome-wide shRNA screening have reported that ADSL is a necessary component for the proliferation of cancerous cells in 40 cell lines. It is notable that approximately 25% of these were breast cancer cell lines, further highlighting the importance of elucidating the role of ADSL in cancer development and progression [34, 35].

Studies investigating the role of ADSL and CYP1A2 SNPs in the development and progression of breast cancer are rather limited. The available data is even scarcer in Central and East Asia, as well as the Middle East, including Iran. Since SNPs are heavily influenced by ethnicity, in this study, we aimed to investigate the role of rs3788579 (ADSL) and rs17861162 (CYP1A2) in female breast cancer patients residing in North-West Iran using PCR–RFLP.

## Materials and method

The current study consists of two separate investigations carried out during the spring and summer of 2020. It was conducted as part of a collaboration between Urmia and Bonab Islamic Azad University, Omid Hospital (Urmia, West Azerbaijan Province, Iran), and Imam Reza Hospital (Tabriz, East Azerbaijan Province, Iran). The study population included 200 female breast cancer patients and 200 healthy women (controls). Whole blood samples (2 cc) were collected from each participant via venipuncture and transferred to sample tubes containing EDTA for DNA and leukocyte extraction.

### DNA Extraction

A DNA extraction kit (EX0061; SINACLON Co., Iran) based on the phenol–chloroform method was used following the manufacturer’s instructions. To ensure DNA quality and quantity, electrophoresis was performed on a 0.7% agarose gel. The A260/A280 ratio, determined using a Nanodrop ND–1000 (Thermo Fisher Scientific, USA), ranged between 1.8–2.0 [36].

### Primer design

The CYP1A2 and ADSL gene sequences were obtained in FASTA format from the GENE database using the NCBI search engine. The mutation loci of ADSL (rs3788579) and CYP1A2 (rs17861162) were obtained from the SNP database using the NCBI search engine (Table 1). The online software Primer 3 was used to determine the REVERSE and FORWARD primer sequences (Table 2).

**Table 1:** Polymorphisms present in human CYP1A2 & ADSL

Gene	Functional consequence	dbSNP (NCBI)	Alleles	Position	Nucleotide change
ADSL	Genic_upstream_transcript_variant,intron_variant	rs3788579	T > A T > C	chr22:40346985 (GRCh38.p14)	CGGGAGCCGCCACCTGTTGCCTCACGTGTTCTCAGTCCGAGAGGGTTCGA GACGCGGAGGAGGCTGGGAGAAATTCAGCCTGTAGTTTCAGGGAATTCAT [T/A/C] TTGGCCATCCCCGCAGGAGTACGCTGCTAACCACAGACACCGAGCGCTTG TTTCGTACTGTTGTGCTCATTATTTCACTAATCTTTTCTTGTGTAATCCA
CYP1A2	3_prime_UTR_variant	rs17861162	C > G	chr15:74756412 (GRCh38.p14)	TCACCATGTTGGCTAGACTAGTCTCAAACTCCTGACCTCAAGTGATCTGC CCGCCTCGACCTCTCTCAAAGTGCTGGCATTACAGGTGTGAGCCACGGTG [C/G] CCGGCCCACAATTAATTTTAGAACATTTTCATCACCCCTAAAAGAAACCC TGCACCCATTAGCAGTCCCTCCACATTTCCCCCTAGCCTGCCTCCCCTGC

**Table 2:** Forward and Reverse primers of CYP1A2 and ADSL genes

Gene		Sequence (5′- > 3′)	Length	Tm	GC%	Self complementarity	Self 3′ complementarity	Product length
CYP1A2	F	GCCTCGACCTCTCTCTCAAAGT	20	59.11	55.00	4.00	1.00	140
	R	GCAGGCTAGGGGGAAATGT	19	59.38	57.89	4.00	2.00	
ADSL	F	GCCTGTAGTTTCAGGGAATTGAT	23	5873	43.48	6.00	2.00	325
	R	CATTCCAGTCCTGTCACTAACT	22	57.46	45.45	4.00	1.00	

### SNP selection and genotyping

The selection of the CYP1A2 and ADSL genes was based on their involvement in metabolic processes. The choice of the ADSL-rs3788579 and CYP1A2-rs17861162 SNPs was made after reviewing available literature from multiple online databases, including PubMed, Google Scholar, Ensembl BioMart, and dbSNP. SNP IDs and frequencies were obtained from sources such as the International 1000 Genomes Project and ENSEMBL BIOMart.

### Polymerase chain reaction (PCR)

The following PCR protocol was used: initial denaturation at 94 °C for 3 min, followed by 40 cycles of denaturation at 94 °C for 30 s, annealing at 55 °C (CYP1A2) and 60°C (ADSL) for 30 s, primer extension at 72 °C for 2 min, and final extension at 72 °C for 10 min. For this purpose, a Thermal cycler (Bio intellectica, Canada) was used. The PCR solution consisted of the following components: 1 µl (≈ 50–100 ng) genomic DNA, 12 µl Taq DNA Polymerase Master Mix (MM2011, SINACLON Co., Iran), 11 µl double-distilled water (ddH2O), and 0.5 µl (≈ 40 pmol) of each forward and reverse primer (Takapou Zist Co., Iran).

### Restriction fragments length polymorphism (RFLP)

The web-based tool NEBCutter v 3.0.17 was used to design RFLP methods. Based on the results, enzymes BSP12861 and SAU3A respectively showed the best cleavage sites at the mononucleotide polymorphism loci of ADSL-rs3788579 and CYP1A2-rs17861162. SNP detection was performed using restriction enzymes according to the manufacturer’s instructions. The chemical digestion reaction was carried out by combining 10 µl of the final PCR solution with 1 µl of BSP 12861 or SAU3A enzymes (CinnaGen Co., Iran), 2 µl of buffer, and 17 µl of sterile distilled water, resulting in a total volume of 30 µl. The process was conducted at 37 °C for 19 h, followed by agarose gel electrophoresis.

### Statistical analysis

Initially, the study utilized the Hardy–Weinberg equilibrium (HWE) to assess allele frequencies for each SNP within the control group. For statistical analysis, the chi-square test and Fisher’s exact test were conducted using SPSS 16.0 software. Genotype analysis considered the homozygous state (AA), heterozygote model (Aa), and additive model (AA vs. Aa vs. aa). The significance level was set at α = 0.05, and the odds ratio (OR) along with a 95% confidence interval (95% CI) was provided.

## Results

**Fig. 1:**
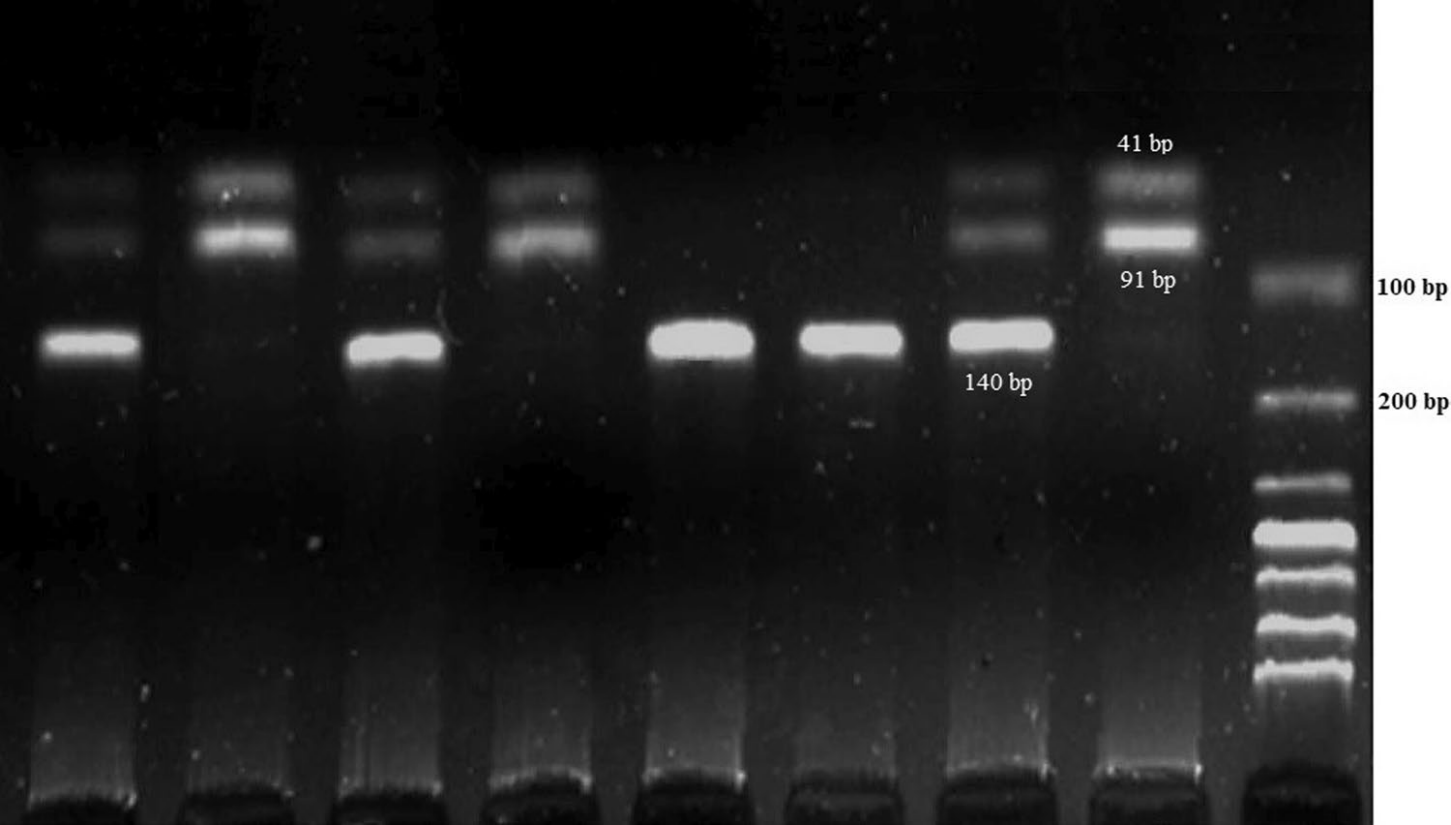
Electrophoresis of products obtained from the enzymatic digestion of the BSP 12861 enzyme

The BSP 12861 enzyme can cleave the wild-type CYP1A2 gene at the rs17861162 locus. Since the enzyme cleaves the G/C at this locus, when the dominant allele (C) is mutated to the disease-related allele (G), BSP 12861 can no longer perform this function. Therefore, BSP 12861 results in the production of a single 140-base pair segment in genes without a splicing locus (mutant homozygous-GG), one 91-base pair and one 49-base pair segments in genes with a single splicing locus (wild-type homozygous-CC), and three segments (140, 91, and 49 base pairs) in genes with two splicing loci (Fig. 1). The CC, CG, and GG genotypes were observed in 54%, 37%, and 9% of the breast cancer patients and 69%, 29%, and 2% of the healthy controls, respectively. In this study, we observed a 21.7% decrease in the CC genotype frequency and a 21.6% and 77.8% increase in the frequencies of the CG and GG genotypes, respectively, compared to controls (P-value ≤ 0.05). The frequency of the C allele was 72.5% and 83.5% in breast cancer patients and controls, respectively. In the same order, the G allele was observed with a frequency of 27.5% and 16.5%. Based on these findings, there may be a possible association between the G allele and an increased risk of developing breast cancer. The risk of breast cancer in patients carrying the G allele was 1.92, which, being greater than one, is associated with an increased risk of developing breast cancer (P-value ≤ 0.05).

In the ADSL-rs3788579 SNP, C and T are the dominant and disease-related alleles, respectively. Since the Sau3a enzyme's splicing locus sequence is GATC/CTAG, the substitution of C with T renders the enzyme ineffective. Hence, enzymatic cleavage results in wild-type homozygous (CC) genes producing three segments (193, 108, and 24 base pairs), and heterozygous (TC) genes produce four segments (193, 192, 108, and 24 base pairs). The 24-base pair segment is a non-polymorphic section of the genome. In our study, the prevalence of the TT, CC, and TC genotypes was 24%, 56%, and 20% in breast cancer patients and 52%, 27%, and 21% in the controls (Table 3) (P-value ≤ 0.05). Among the healthy participants, genotypes CC and TC were the most and least frequent, respectively. In contrast, genotypes TT and TC were the most and least frequent genotypes in the breast cancer group. Based on the Chi-square test, the difference in genotype distribution between the two groups was significant. The T allele is the most frequent allele in breast cancer patients; compared to the healthy controls, this allele was 28.5% more frequently observed in the breast cancer group. We suggest a possible link between the higher frequency of the T allele and the development of breast cancer. Since carrying the T allele was associated with a 3.235-fold risk of breast cancer, this risk factor possibly increases the likelihood of breast cancer (P-value ≤ 0.05). In this study, both groups conform to the Hardy–Weinberg principle.

**Table 3:** Association of CYP1A2 and CYP3A4 genetic polymorphisms

Gene	dbSNP	Genotype	Cancer (%)	Control (%)	P-Value	OR (95% CI)
ADSL	rs3788579	TT	52	24	Ref	Ref = 1
		CC	27	56	0.000	1.788 (1.451–2.202)
		CT	21	20	0.000	0.553 (0.445–0.686)
CYP1A2	rs17861162	CC	54	69	Ref	Ref = 1
		GG	9	2	0.022	0.70 (0.52–0.93)
		CG	37	29	0.026	1.34 (1.10–1.64)

Gene	allele	Cancer (%)	Control (%)	P-Value	OR (95% CI)
ADSL	C	37.5	66.0	Ref	Ref = 1
	T	62.5	34.0	0.000	3.235 (2.149–4.872)
CYP1A2	C	72.5	83.5	Ref	Ref = 1
	G	27.5	16.5	0.008	1.92 (1.18–3.11)

Pationts: 100, Controls: 100, P-Value ≤ 0.05

The study investigated the influence of two SNPs in the ADSL and CYP1A2 genes on clinicopathological parameters in patients, including menstrual status and age at diagnosis (Table 4). In the case of CYP1A2 rs17861162, individuals with the CC genotype were more likely to be of older age compared to carriers with the combined genotype (GG + CG), while the opposite was observed for pre-menopausal status. There were no identified significant correlations between the genotypes of CYP3A4 rs3788579 and the clinicopathological parameters (P-value ≤ 0.05).

**Table 4:** Association between the clinicopathological variables and the genotypes of CYP1A2 and CYP3A4 genetic polymorphisms

Factor		CYP1A2 (rs17861162)		P-value	ADSL (rs3788579)		P-value
		CC %	CG + GG %		TT %	CC + CT %	
Age (years)	< 50	32	26	0.021	30	27	0.651
	≥ 50	28	14		23	20	
Menopause status	Pre-menopause	31	26	0.001	30	27	0.517
	Post-menopause	30	13		24	19	

Pationts: 100, P-Value ≤ 0.05

## Discussion

Breast cancer is one of the most common malignancies in women, with over 1 million new cases diagnosed annually. Recent research indicates a growing prevalence of breast cancer among Iranian women, particularly at a younger age, which raises concerns [37, 38]. Although our knowledge of breast cancer is still evolving, we have identified various genetic risk factors associated with the disease [10].

Our current study's results reveal that CYP1A2 SNPs (rs17861162) in breast cancer patients are associated with a 21.7% decrease in the frequency of the CC genotype and a 21.6% and 77.8% increase in the frequency of CG and GG genotypes, respectively. Furthermore, this SNP leads to an 11% decrease in the frequency of the C allele and an 11% increase in the frequency of the G allele within the breast cancer group compared to healthy controls (P-value = 0.00). Altogether, the CYP1A2 SNP (rs17861162) results in an alteration of the allelic balance observed in women without breast cancer. Notably, there is a limited body of research on the impact of the rs17861162 locus polymorphism on the CYP1A2 gene, and as far as our knowledge extends, no previous study has explored this aspect in Iran.

The known and possible functional roles of this SNP in breast cancer have not yet been revealed precisely, however, CYP1A2 plays a prominent role in the activation of pre-carcinogens through metabolic processes [39] and SNPs can alter the activity of the corresponding proteins [19]. Given the prominent role of human Cytochrome P450 enzymes in the metabolism of medications, toxins, and chemical compounds, any alteration to the genomic sequence encoding these enzymes may subsequently elevate the risk of cancer [18, 42]. It is noteworthy that the link between clinicopathological characteristics and the prognosis of breast cancer with the CYP1A2-rs17861162 SNP was first identified by Bai et al. They reported a significant correlation between the CYP1A2-rs17861162 SNP and factors such as age, menstrual status, and the presence of the P53 marker in women diagnosed with breast cancer. In comparison to individuals with the CC genotype, those carrying this SNP, particularly the combined genotype (GG + CG), were more likely to be younger and pre-menopausal, as well as to test negative for the P53 tumor marker [10]. CYP1A2 acts as an agent for metabolizing endogenous compounds such as steroid hormones, retinoic acid, and bile acids [39]. Estrogen levels significantly influence breast cancer risk in postmenopausal women, a factor intricately linked to both menstrual status and age [40]. However, Bai et al. did not observe any connection between this SNP and the patient's genotype in relation to breast cancer prognosis. Additionally, the interplay between coffee consumption and the CYP1A2*1F genotype has been shown to impact the age at which breast cancer is diagnosed and the status of estrogen receptors [41].

Khvostova et al. investigated the polymorphism of estrogen-metabolizing enzymes (cytochromeP450s: CYP1A1, CYP1A2, and CYP19) in breast cancer patients within a cohort of Siberian women. They reported several polymorphisms associated with an increased risk of cancer development and progression. Furthermore, they observed that the CYP1A2 polymorphism increases the risk of developing estrogen receptor-positive tumors [43]. Liu et al. observed that SNPs occurring in CYP1A2-rs17861162 and –rs-11636419 influence the efficacy of epidural ropivacaine in women with breast cancer [44]. Ghotbi et al. reported differences in CYP1A2 polymorphism frequencies between the Swedish and Korean populations [45], while Lim et al. observed significant inter-ethnic differences among the Chinese, Malay, and Indian populations [22]. Perera et al. observed a significant difference in CYP1A2 activity between South Asian and European ethnicities [23]

CYP1A2 plays a pivotal role in the metabolism of 10–13% of all carcinogens and medications, including clozapine, olanzapine, theophylline, and heterocyclic aromatic amines [22, 23, 46]. It is also involved in metabolizing substances like coffee and estrogen [47], while inactivating exogenous compounds such as environmental pro-carcinogens [10]. CYP1A2 significantly contributes to the 2-hydroxylation of the primary estrone and estradiol [47, 48]. Notably, through the activation of various carcinogenic heterocyclic amines, CYP1A2 may contribute to the progression of specific malignancies [22]. The literature underscores that genetic polymorphisms substantially influence the expression and activity of CYP1A2 [10, 24]. To date, over 30 SNPs have been identified in the CYP1A2 upstream sequence and its intron-1 region [23], contributing to its role in cancer development and progression [10, 49].

The link between CYP1A2 polymorphisms and an increased susceptibility to developing lung, colorectal, breast, and biliary cancers has been established [43, 50]. In general, the role of CYP1A2 polymorphisms in cancer development remains a subject of debate. While numerous studies have investigated the role of CYP1A2 polymorphisms in breast cancer development among different ethnicities, findings have not consistently aligned [10, 47]. Some studies report a negative association between CYP1A2*1F and breast cancer incidence [25] or that the presence of the C allele is protective against the disease [25, 51]. However, others have failed to observe the same trends [25, 48] or establish their precise role in disease development [48]. Additional investigations have reported associations between CYP1A2 SNPs and breast cancer [10]. Increased CYP1A2 gene activity has also been linked to a heightened risk of breast cancer development [52]. These discrepancies may arise from various individual, genetic, and ethnic determinants, alongside exposure to environmental risk factors and differences in healthcare approaches or a complex interplay between these factors [22].

Additionally, in our investigation of the ADSL gene's SNP rs3788579, we found a significantly higher prevalence of the T allele in individuals with breast cancer compared to the healthy group, exceeding by 28.5% (P-value = 0.00). This finding suggests a potential association between the increased frequency of the T allele and breast cancer incidence. Furthermore, carriers of the T allele exhibited a 3.235-fold higher risk of developing breast cancer, indicating that the presence of this allele may contribute to an elevated risk of breast cancer incidence. To the best of our knowledge, no previous investigation has been made into the impact of ADSL-rs3788579 SNP on breast cancer development.

The ADSL gene exhibits typical characteristics of a housekeeping gene [53]. Functioning as an essential homotetrameric enzyme, ADSL plays a pivotal role in two key reactions: 1) the conversion of succinylaminoimidazolecarboxamide (SAICA)-ribotide (SAICAR) into AICA-ribotide (AICAR) through the de novo purine synthesis pathway, and 2) the generation of AMP by converting adenylo-succinate into adenosine monophosphate as part of the purine nucleotide cycle [54]. Potential modifications to the purine nucleotide degradation pathway resulting from non-synonymous SNPs in the ADSL gene could lead to structural or functional alterations in its associated protein [55]. Available evidence suggests that point mutations within the ADSL gene can result in significant variations in IMP content [56, 57]. Chen et al. investigated the relationship between the 7 SNP genotypes and expression levels of the ADSL gene in CEU cell lines, finding significant correlations between ADSL-rs8135371 and -17001863 and the expression of the ADSL gene. Furthermore, the total copies of the C and G alleles were found to increase ADSL expression in rs8135371 and rs17001863 SNPs, respectively [58].

The ADSL gene plays a crucial role in maintaining the ATP/AMP ratio, a fundamental factor in regulating cell division and metabolism [53]. It contributes to this equilibrium by providing the necessary purine nucleotides required for DNA replication and cell division. In vertebrates, the purine nucleotide degradation pathway consists of ten enzymatic steps, with ADSL, AMPD1, and ATIC being among the most significant. As ADSL oversees two of these pathway steps, modifications to the enzyme at either of these stages can lead to subsequent alterations in IMP content [53].

Increased levels of ADSL have been observed in various conditions, including colorectal, breast, and prostate cancer. Furthermore, genomic variations have been associated with specific traits, such as drug responsiveness, along with changes in gene expression [58]. Despite the recognized disruption of ADSL activity in various malignancies, such as breast, colorectal, prostate cancer, tubulovillous and tubular adenoma, glioma, and more, the precise mechanisms governing the impact of ADSL on the initiation and progression of these disorders remain incompletely understood [34, 59, 60].

Zurlo et al. documented that depletion of ADSL leads to an increase in the expression of the long non-coding RNA MIR2HG by altering adenosine and adenine levels. MIR22HG, in turn, reduces the expression of the oncogene c-YMC at the protein level. Consequently, knocking out ADSL hampers the growth and invasion of triple-negative breast cancer (TNBC) cells, both in vitro and in vivo [34]. Park et al. proposed that the ADSL gene promotes oncogenesis in endometrial cancer by upregulating the expression of killer cell lectin-like receptor C3 (KLRC3) through fumarate production. Their findings indicated that ADSL enhances tumor cell proliferation, invasion, and migration by modulating KLRC3 expression. The application of external fumarate restored KLRC3 expression in ADSL knockout cells, indicating that ADSL regulates KLRC3 expression through fumarate production [59]. Evidence confirms that in human endometrioid carcinoma, ADSL expression was correlated with increased histological aggressiveness and the extent of primary tumor progression.

Breast cancer arises from the accumulation of genetic defects in epithelial cells, resulting in the presentation of malignant phenotypes [61]. Although novel therapeutic options have significantly improved breast cancer treatment, the inherent histological, molecular, and genomic heterogeneity of this malignancy complicates gene-based interventions [62]. Due to the limitations and disadvantages of conventional diagnostic modalities such as X-rays, Ultrasound, and Computed Tomography (CT) scans, less invasive and cost-effective alternatives are gaining popularity. In this context, screening for genetic markers, such as CYP1A2 and ADSL, is considered a potential alternative to conventional methods. Numerous genomic variants, also known as SNPs, including CYP1A2 (rs17861162) and ADSL (rs3788579), appear to be correlated with the risk of breast cancer development. Investigating these SNPs may contribute to improving diagnostic accuracy due to their significant interpersonal and genetic variations [63, 64]. Besides their diagnostic potential, these markers may be useful in the early detection of breast cancer and risk assessment for individuals with this malignancy.

## Conclusion

In conclusion, our findings demonstrate a notable shift in allele frequencies within the CYP1A2-rs17861162 SNP, from the prevalent C allele to the disease-associated G allele, resulting in an 11% increase, as well as a substantial 28.5% increase in the T allele of the ADSL-rs3788579 SNP among female breast cancer patients in North-West Iran when compared to healthy controls. These significant genetic alterations strongly suggest their potential association with the development and progression of breast cancer within this specific population. This underscores the promising utility of CYP1A2-rs17861162 and ADSL-rs378879 SNP detection as potential diagnostic and prognostic biomarkers.

## Data Availability

The datasets produced and/or analyzed in this study can be obtained from the corresponding author upon reasonable request.

## References

[1] Wilkinson L, Gathani T (2022). Understanding breast cancer as a global health concern. Br J Radiol.

[2] Jassim GA, Doherty S, Whitford DL, Khashan AS (2023). Psychological interventions for women with non-metastatic breast cancer. Cochrane Database Syst Rev.

[3] Nolan E, Lindeman GJ, Visvader JE (2023). Deciphering breast cancer: from biology to the clinic. Cell.

[4] Trayes KP, Cokenakes SEH (2021). Breast cancer treatment. Am Fam Physician.

[5] Britt KL, Cuzick J, Phillips K-A (2020). Key steps for effective breast cancer prevention. Nat Rev Cancer.

[6] Giaquinto AN (2022). Breast cancer statistics, 2022. CA Cancer J Clin.

[7] Fouladi N, Pourfarzi F, Amani F, Ali-Mohammadi H, Lotfi I, Mazaheri E (2012). Breast cancer in Ardabil province in the north-west of Iran: an epidemiological study. Asian Pac J Cancer Prev.

[8] Rezagholi T (2015). Estimating the burden of breast cancer in Iranian women in 2009. Iran J Epidemiol.

[9] Aghoosi H, Majid S, Nabatchian F, Mordadi A, Khodaverdi F (2015). Evaluation of effects of alfalfa extract and risk of breast cancer. J Payavard Salamat.

[10] Bai X, Xie J, Sun S, Zhang X, Jiang Y, Pang D (2017). The associations of genetic polymorphisms in CYP1A2 and CYP3A4 with clinical outcomes of breast cancer patients in northern China. Oncotarget.

[11] Deng C-X (2003). Roles of BRCA1 in DNA damage repair: a link between development and cancer. Hum Mol Genet.

[12] Claus EB, Schildkraut JM, Thompson WD, Risch NJ (1996). The genetic attributable risk of breast and ovarian cancer. Cancer.

[13] Sheweita S, Tilmisany A (2003). Cancer and phase II drug-metabolizing enzymes. Curr Drug Metab.

[14] Bond GL, Hu W, Levine A (2005). A single nucleotide polymorphism in the MDM2 gene: from a molecular and cellular explanation to clinical effect. Cancer Res.

[15] Wolff MS, Weston A (1997). Breast cancer risk and environmental exposures. Environ Health Perspect.

[16] Hashemi M (2013). Functional polymorphisms of FAS and FASL gene and risk of breast cancer–pilot study of 134 cases. PLoS ONE.

[17] Ioannides C (2008). Cytochromes P450: role in the metabolism and toxicity of drugs and other xenobiotics.

[18] Lamba JK, Lin YS, Schuetz EG, Thummel KE (2012). Genetic contribution to variable human CYP3A-mediated metabolism. Adv Drug Deliv Rev.

[19] Carney SA, Peterson RE, Heideman W (2004). 2, 3, 7, 8-Tetrachlorodibenzo-p-dioxin activation of the aryl hydrocarbon receptor/aryl hydrocarbon receptor nuclear translocator pathway causes developmental toxicity through a CYP1A-independent mechanism in zebrafish. Mol Pharmacol.

[20] Landi MT, Sinha R, Lang NP, Kadlubar FF (1999). Human cytochrome P4501A2. IARC Sci Publ.

[21] Bernauer U, Heinrich-Hirsch B, Tönnies M, Peter-Matthias W, Gundert-Remy U (2006). Characterisation of the xenobiotic-metabolizing Cytochrome P450 expression pattern in human lung tissue by immunochemical and activity determination. Toxicol Lett.

[22] Lim JSL (2010). Pharmacogenetics of CYP1A2, novel polymorphisms and haplotypes in three distinct Asian populations. Drug Metab Pharmacokinet.

[23] Perera V, Gross AS, McLachlan AJ (2012). Influence of environmental and genetic factors on CYP1A2 activity in individuals of South Asian and European ancestry. Clin Pharmacol Ther.

[24] Dobrinas M, Cornuz J, Oneda B, Serra MK, Puhl M, Eap CB (2011). Impact of smoking, smoking cessation, and genetic polymorphisms on CYP1A2 activity and inducibility. Clin Pharmacol Ther.

[25] Le Marchand L, Donlon T, Kolonel LN, Henderson BE, Wilkens LR (2005). Estrogen metabolism–related genes and breast cancer risk: the multiethnic cohort study. Cancer Epidemiol Prevent Biomarkers.

[26] Tabucchi A, Carlucci F, Rosi F, Guerranti R, Marinello E (2001). Determination, activity and biological role of adenylosuccinate lyase in blood cells. Biomed Pharmacother.

[27] Pedley AM, Benkovic SJ (2017). A new view into the regulation of purine metabolism: the purinosome. Trends Biochem Sci.

[28] Zhu R (2017). ADSL, AMPD1, and ATIC expression levels in muscle and their correlations with muscle inosine monophosphate content in dapulian and hybridized pig species. Open J Anim Sci.

[29] Du Wenxing XSYD (2011). Current research advances in IMP and related enzymes. Biotechnol Bull.

[30] Yuan T, Gu J-R, Gu W-B, Wu J, Ge S-R, Xu H (2011). Molecular cloning, characterization and expression analysis of adenylosuccinate lyase gene in grass carp (Ctenopharyngodon idella). Mol Biol Rep.

[31] Natsumeda Y (1985). Purine enzymology of human colon carcinomas. Cancer Res.

[32] Reed VL, Mack DO, Smith LD (1987). Adenylosuccinate lyase as an indicator of breast and prostate malignancies: a preliminary report. Clin Biochem.

[33] Jurecka A, Zikanova M, Kmoch S, Tylki-Szymańska A (2015). Adenylosuccinate lyase deficiency. J Inherit Metab Dis.

[34] Zurlo G (2019). Prolyl hydroxylase substrate adenylosuccinate lyase is an oncogenic driver in triple negative breast cancer. Nat Commun.

[35] McDonald ER (2017). Project DRIVE: a compendium of cancer dependencies and synthetic lethal relationships uncovered by large-scale, deep RNAi screening. Cell.

[36] Dilhari A (2017). Evaluation of the impact of six different DNA extraction methods for the representation of the microbial community associated with human chronic wound infections using a gel-based DNA profiling method. AMB Express.

[37] Taghavi A (2012). Increased trend of breast cancer mortality in Iran. Asian Pac J Cancer Prev.

[38] Mousavi SM (2007). Breast cancer in Iran: an epidemiological review. Breast J.

[39] Rendic S (2002). Summary of information on human CYP enzymes: human P450 metabolism data. Drug Metab Rev.

[40] Fuhrman BJ (2012). Estrogen metabolism and risk of breast cancer in postmenopausal women. J Natl Cancer Inst.

[41] Bågeman E, Ingvar C, Rose C, Jernström H (2008). Coffee consumption and CYP1A2* 1F genotype modify age at breast cancer diagnosis and estrogen receptor status. Cancer Epidemiol Biomark Prev.

[42] Coley HM (2008). Mechanisms and strategies to overcome chemotherapy resistance in metastatic breast cancer. Cancer Treat Rev.

[43] Shan J (2012). Genetic polymorphism of estrogen metabolizing enzymes in Siberian women with breast cancer. Genet Test Mol Biomarkers.

[44] Liu J, Xi H, Jiang Y, Feng Z, Hou L, Li W (2015). Association of CYP450 single nucleotide polymorphisms with the efficacy of epidural ropivacaine during mastectomy. Acta Anaesthesiol Scand.

[45] Ghotbi R, Christensen M, Roh H-K, Ingelman-Sundberg M, Aklillu E, Bertilsson L (2007). Comparisons of CYP1A2 genetic polymorphisms, enzyme activity and the genotype-phenotype relationship in Swedes and Koreans. Eur J Clin Pharmacol.

[46] Zhou S, Wang B, Yang L, Liu J-P (2010). Structure, function, regulation and polymorphism and the clinical significance of human cytochrome P450 1A2. Drug Metab Rev.

[47] Bageman E, Ingvar C, Rose C, Jernstrom H (2008). Coffee consumption and CYP1A2*1F genotype modify age at breast cancer diagnosis and estrogen receptor status. Cancer Epidemiol Biomark Prev.

[48] Long J-R (2006). Population-based case??? control study of AhR (aryl hydrocarbon receptor) and CYP1A2 polymorphisms and breast cancer risk. Pharmacogenet Genomics.

[49] Božina N, Bradamante V, Lovrić M (2009). Genetic polymorphism of metabolic enzymes P450 (CYP) as a susceptibility factor for drug response, toxicity, and cancer risk. Arch Ind Hyg Toxicol.

[50] Xiao-Feng H (2014). Association between CYP1A2 and CYP1B1 polymorphisms and colorectal cancer risk: a meta-analysis. PLoS One..

[51] Kotsopoulos J (2007). The CYP1A2 genotype modifies the association between coffee consumption and breast cancer risk among BRCA1 mutation carriers. Cancer Epidemiol Prevent Biomarkers.

[52] Hong C-C, Tang B-K, Hammond GL, Tritchler D, Yaffe M, Boyd NF (2004). Cytochrome P450 1A2 (CYP1A2) activity and risk factors for breast cancer: a cross-sectional study. Breast Cancer Res.

[53] Yan J (2018). Effects of exogenous inosine monophosphate on growth performance, flavor compounds, enzyme activity, and gene expression of muscle tissues in chicken. Poult Sci.

[54] Camici M (2023). Inborn errors of purine salvage and catabolism. Metabolites.

[55] Lim D (2016). Analysis of extended haplotype in Korean cattle (Hanwoo) population. BMB Rep.

[56] Gong LL (2011). Genetic variation and expression of ADSL and GARS-AIRS-GART genes and their associations with meat quality in chicken.

[57] Ye MH, Chen JL, Zhao GP, Zheng MQ, Wen J (2010). Correlation between polymorphisms in ADSL and GARS-AIRS-GART genes with inosine 5′-monophosphate (IMP) contents in Beijing-you chickens. Br Poult Sci.

[58] Chen S-H (2011). A genome-wide approach identifies that the aspartate metabolism pathway contributes to asparaginase sensitivity. Leukemia.

[59] Park H (2018). Adenylosuccinate lyase enhances aggressiveness of endometrial cancer by increasing killer cell lectin-like receptor C3 expression by fumarate. Lab Invest.

[60] Jiang T (2021). Targeting de novo purine synthesis pathway via ADSL depletion impairs liver cancer growth by perturbing mitochondrial function. Hepatology.

[61] Melville S, Heycock L (2007). Breast cancer: an overview. Pharm J.

[62] Álvarez RH (2010). Present and future evolution of advanced breast cancer therapy. Breast Cancer Res.

[63] Beck T, Hastings RK, Gollapudi S, Free RC, Brookes AJ (2014). GWAS Central: a comprehensive resource for the comparison and interrogation of genome-wide association studies. Eur J Hum Genet.

[64] Shan J (2012). Genome-wide association studies (GWAS) breast cancer susceptibility loci in Arabs: susceptibility and prognostic implications in Tunisians. Breast Cancer Res Treat.

